# The Taste of Commercially Available Clarithromycin Oral Pharmaceutical Suspensions in the Palestinian Market: Electronic Tongue and In Vivo Evaluation

**DOI:** 10.3390/s18020454

**Published:** 2018-02-03

**Authors:** Nawaf Abu-Khalaf, Abdel Naser Zaid, Nidal Jaradat, Alaaldin AlKilany, Basima Abu Rumaila, Rowa Al Ramahi, Shrouq Shweiki, Safaa Nidal, Nibal Surakhi

**Affiliations:** 1 College of Agricultural Sciences and Technology, Palestine Technical University-Kadoorie (PTUK), Tulkarm P.O.Box 7, Palestine; nawafu@hotmail.com (N.A.-K.); basima.aaar.90.bsm@gmail.com (B.A.R.); 2 Department of Pharmacy, Faculty of Medicine and Health Sciences, An-Najah National University, Nablus P.O.Box 7, Palestine; nidaljaradat@najah.edu (N.J.); A.AlKilany@ju.edu.jo (A.A.); rawa_ramahi@najah.edu (R.A.R.); shoruq_shweiki94@hotmail.com (S.S.); safaa.nidal.94@gmail.com (S.N.); surakhinibal@gmail.com (N.S.); 3 School of Pharmacy, Jordan University, Amman 11942, Jordan

**Keywords:** alpha-astree, electronic tongue, clarithromycin, taste, pediatric

## Abstract

**Background**: The taste of oral liquid dosage forms is a crucial factor that impacts paediatric patient compliance. The electronic tongue (ET) is an emerging tool that could be useful in taste assessment in order to minimize the involvement of humans in such evaluations. **Purpose**: The aim of this study is to evaluate the taste of commercially available clarithromycin (CM) oral pharmaceutical suspensions in the Palestinian market. **Method**: Commercially available CM suspensions (the brand Klacid^®^ and two generic K1 and K2) were assayed using the high performance liquid chromatography (HPLC) method. Then, the taste of these products was assessed using alpha-astree ET. In addition, an in vivo taste assessment was conducted on paediatric patients by a hedonic panel test. Moreover, volunteering community pharmacists were asked to rank the taste of these three products according to their experience from the best to the worst. **Results**: All suspension products had a CM concentration not less than 98% of the label amount. The ET results coupled with the principal component analysis (PCA) showed a very clear discrimination of the samples with different distances between groups (*p*-values < 0.001). Suspensions were in the following order in terms of taste: Klacid^®^ > K1 > K2. Moreover, The pattern discrimination index between (K1 and Klacid^®^), (K1 and K2) and (Klacid^®^ and K2) were 8.81%, 65.75%, and 71.94%, respectively which suggests that K1 and Klacid^®^ are the most similar preparations in terms of taste. Interestingly, these results were in excellent agreement with the pharmacist ranking and patient acceptance test. **Conclusions**: The evaluated preparations showed significantly different taste within the order of Klacid^®^ > K1 > K2, as suggested by both the ET and in vivo results. Moreover, our results confirm the capability of alpha-astree ET in the taste assessment of oral suspensions and in predicting volunteer responses, which highlights its beneficial use as an in vitro taste assessment tool and as an alternative to human-based taste evaluations.

## 1. Introduction

Organoleptic properties, especially taste, are considered a serious challenge during the development of various oral dosage forms. In fact, the taste of an oral drug product plays a crucial role in patient compliance and consequently therapeutic effectiveness. Thus an increasing interest in taste evaluation and masking has been witnessed, with increasing attention from regulatory authorities as well [1]. Traditionally, the assessment of pharmaceutical product taste is usually conducted with human volunteers. However, this may be costly, time-consuming and sometimes harmful, if the drug has a low therapeutic index or serious side effects. Accordingly, reliable and predictive in vitro methods have been developed to evaluate pharmaceutical product taste [2,3,4,5,6,7]. One of the most commonly used methods is the electronic tongue (ET) device, which is a multi-sensor system that consists of a number of low-selective and cross-sensitive sensors, and uses advanced mathematical procedures for signal processing based on multivariate analysis, e.g., pattern recognition (PARC) and artificial neural networks (ANNs) [8,9,10,11]. ET is a robust device that showed a fast determination of several compounds in the solution and a sample classification with a direct measuring stage. In addition, it does not require sample preparation and the same samples can be measured several times since there is no change in their characteristics after the first trial of measurements [12,13,14]. The ET has been used in: (i) environmental and biotechnology applications, (ii) the food industry, (iii) olive oil authentication and adulteration, and most importantly (iv)oral pharmaceutical product evaluation [13,15,16,17,18,19,20,21,22,23,24]. The ET has been employed to evaluate the taste of a wide array of pharmaceuticals and has been proven to be a useful tool in dosage form development (taste attributes), comparison of various products (competitors) and taste-masking initiatives. Critical and comprehensive reviews of the application of ET in the pharmaceutical field are available in the literature [25,26]. In fact, ET is becoming an important tool in pharmaceutical development to assess the final taste of oral pharmaceutical products. Recently, a successful qualification, according to the international conference on harmonization (ICH), of the use of ET has been reported [27].

Clarithromycin (CM) is used to treat several types of bacterial infections affecting the skin, urinary andrespiratory system. It is also used in combination with other medicines (triple therapy) to treat stomach ulcers caused by Helicobacter pylori [28]. This drug is commercially available in various dosage forms, including suspension for paediatric use.

It is a white powder, with a water solubility around 0.33 mg/L and a very strong bitter taste. In fact, due to this unpleasant bitter taste, the development of a tasty oral paediatric CM suspension represents a huge problem and challenge during formulation. Accordingly, the crystals of this drug are previously coated in order to mask their bitter taste. Despite this pharmaceutical procedure and the addition of sweetening and flavouring agents, this undesired taste remains clearly in the mouth resulting in poor patient compliance; this may result in serious difficulties in patient treatment and bacterial resistance. In addition, the in vivo assessment of the taste of the suspension during pre-formulation, formulation and scale up would be costly, very difficult and impractical, especially in the absence of expert paediatricpanelists [29]. Uchida and co-workers reported the capability of ET to unveil the complete masking of the CM bitter taste in a commercial clarithromycin dry syrup product (Clarith dry syrup, Taisho Pharmaceutical Co. Ltd., Tokyo, Japan) that has granules coated with acrylate-based polymer to mask the taste. The taste sensor results confirmed that the polymer was successful in almost completely masking the bitter taste of the dry syrup product [30]. This study aimed at evaluating the taste of commercially available CM oral pharmaceutical suspensions*brand and two generics) in the Palestinian market by ET and volunteer panel consistingofpaediatric patients and community pharmacists.

## 2. Materials and Methods

### 2.1. Formulations

Klaricare^®^ was produced by Pharmacare PLC (Ramallah, Palestine), Klarimax^®^ was produced by BeitJala pharmaceutical company (BeitJala, Palestine) and the brand, Klacid^®^, was manufactured by Abbott Company (New York, NY, USA). All these CM paediatric oral suspensions (250 mg/5 mL) were bought from a community pharmacy (University Pharmacy, Nablus, Palestine), used as per label and used before expiration dates.

### 2.2. Chemical and Reagents

United state Pharmacopoeia (USP) CM reference standard (RIs; Holland Moran, Holon, Israel) was used. High-performance liquid chromatography (HPLC)-grade solvents such as acetonitrile and methanol were purchased from EMD Millipore (Billerica, MA, USA) and were used as received. Potassium dihydrogen phosphate, sodium hydroxide pellets, phosphoric acid, triethylamine, and glacial acetic acid were also purchased from EMD Millipore. Purified water was obtained using an EMD Millipore Milli-Q plus water purification system. 

### 2.3. Instruments

The HPLC system (Merck–Hitachi, Kent, UK) equipped with model L-7100 pump, L-7200 autosampler, L-7300 column oven, DAD L-7450 photodiode array detector, and D-7000 software HSM Version 3.1 (Merck–Hitachi). The HPLC experimental conditions were optimized on a theoctadecylsilane C18 chemically bonded column (125 × 3.0 mm id, 5 mcm particles) that was purchased from ACE (London, UK). Weights were measured using Ohaus balance (Model DV215CD; Shekel Ltd., Petah-Tikva, Israel); the pH was identified using Toledo GmbH pH meter (Model S47-K; Agentek, Mettler Toledo, Greifensee, Switzerland).

An alpha-astree ET device (Alpha MOS, Toulouse, France) was used for the experiment. It is composed of a sensor array of seven sensors, and their measurements are based on the potentiometric principle. The ET was equipped with a 16-position auto-sampler, an automatic stirrer, and an Ag/AgCl reference electrode. The device has a software package for multivariate data analysis (chemometrics) named AlphaSoft software (Alpha MOS, Toulouse, France), which also automatically collected and stored the sensors’ outputs signal (Alpha MOS, 2009).

### 2.4. Methods

#### 2.4.1. Quality Control of Evaluated Suspensions

The brand and generic formulations were assessed for the list of used inactive ingredients. In fact, they were found to contain carbopol 974P, povidone K90, hypromellose phthalate (HP-55), castor oil, silicon dioxide, sucrose, xanthan gum, flavour—fruit punch, potassium sorbate, citric acid, titanium dioxide, sodium chloride and maltodextrin.Visual examination was used to assess the appearance of the reconstituted suspensions. The viscosity of the suspension was measured for all the formulations using a Brookfield viscometer (Brookfield, New York, NY, USA) using spindle number three and the rotation speed was 30 rounds per minute. pH measurements were obtained for reconstructed suspensions (125 mg/5 mL) with purified water at room temperature using a sevenmulti pH meter (MettlerToledo, Columbus, OH, USA). 

The CM assays were carried outaccording to the procedure reported in the USP using the HPLC method (optical absorption at 218 nm). The flow rate was 1.1 mL/min and the running time was 15 min [31]. Monobasic potassium phosphate buffer (0.067 M) was prepared. The mobile phase consisted of a mixture of methanol and buffer (3:2) and the pH was adjusted using phosphoric acid to a pH of 3.5. The standard stock solution was prepared by dissolving CM powder in methanol in order to contain an equivalent to 2.1 mg/mL. The standard solution was prepared to contain 0.415 mg/mL of CM from standard stock solution in the themobile phase.

To prepare the sample stock solution, CM oral suspension was reconstituted as directed by the label of the drug product. Precisely, an aliquot of the suspension equivalent to 1–2 g of CM, with the aid of 330 mL of buffer, was transferred to a 1000-mL volumetric flask containing 50 mL of the buffer. The suspension was shacked vigorously for 30 min, the final volume was adjusted using methanol, and the obtained suspension was sonicated for about 30 min to ensure complete dissolving of CM. After cooling, the suspension was allowed to settle, and a clear supernatant was used. Regarding the preparation of the sample solution, an aliquot of the sample stock solution, nominally equivalent to 20 mg of CM was transferred to a 50-mL volumetric flask, and then diluted up to volume with the same mobile phase. 

#### 2.4.2. In Vitro Assessment of Taste Using ET

Before samples were analyzed, the seven sensors went through a conditioning, calibration, and diagnostic process according to manufacturer’s recommendation. Cleaning of the sensor array was carried out between each measurement using pure distilled water (Alpha MOS, 2009). In addition, data reduction (i.e., scaled and normalized) was conducted before the PCA was performed. Three samples of CM were re-suspended in 100 mL distilled water to yield asimilarconcentration of 25 mg/5 mL. Each sample was measured in triplicate. Data acquisition and data processing were achieved with AlphaSoft software. Multivariate data analysis was used to analyze data. Before the beginning of each measurement, a short run with three different concentrations (low, medium and high) of CMwas performed for the conditioning of the sensors. This fast preliminary test was conducted to check the sensitivity of the sensor toward the tested products. However, the results clarified that the ET was sensitive to the compounds with their tested concentration.

#### 2.4.3. In Vivo Assessment of Taste

This study was divided into two parts: the first part was conducted on paediatric patients and the second part was conducted on pharmacists. The study proposal was approved by the Institutional Review Board (IRB) of An-Najah National University. The protocol of the study was initially prepared and submitted to the IRB according to good clinical practice (GCP) guidelines [32,33]. 

##### Study Design and Inclusion Criteria

The first part of this study included paediatric patients aged 3–12 years who were prescribed one of the commercial CM suspensions registered in the Palestinian market and who donot have an allergy to CM or one of theproduct’s ingredients. The nature and importance of this study were explained to the parents who came to the community pharmacy and they were asked to approve the enrolment of their children in the study. Their verbal consent was obtained [34]. 

For the second part of this study, 150 community pharmacists were invited to evaluate the taste of CM oral suspensions available in the Palestinian market based on accumulated feedback from parents along with their experience. These pharmacists were asked to rank these oral suspensions according to their taste (from the best to the worst). In addition, they were also asked to rank this suspension according to their prescriptions (from the most prescribed to the least).

##### Data Collection and Statistical Analysis

**Figure 1:**
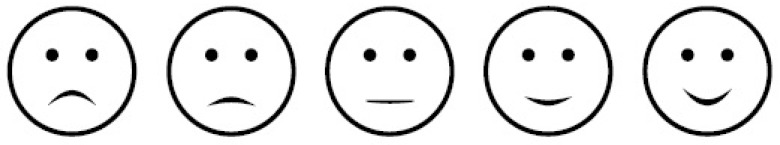
Hedonic scale.

All products were tasted in the same manner. Precisely, a teaspoonful of the prescribed CM suspension was administered to each paediatric patient and then the participants were asked to immediately document their overall perception of the taste of the administered product using a hedonic scale, in which products were ranked according to a numerical scale (one to five) where one indicates a very sad and unacceptable taste, while five indicates a happy face due to the good taste of the product (Figure 1) [33]. 

Data wereanalyzed using statistical package for Social Sciences Software (SPSS 22). Categorical variables were expressed as frequencies and percentages. The mean ± standard deviation was computed for continuous data. Frequencies and percentages were calculated for categorical variables. The Kolmogorov–Smirnov test was used to evaluate the normal distribution of the variables and the Kruskal–Wallis test was used according to that. A probability (*p*) value of less than 0.05 was considered to be statistically significant for all analyses.

## 3. Results

### 3.1. Visual and Pharmacopoeial Assessment

The visual examination of the reconstituted suspensions showed no defects in any of the examined products. They showed similar viscosity and pH when compared with the reference listed drug (RLD). All suspension products had a CM content not less than 98% of the label amount. This suggests that the products were similar in their drug content and the results of the taste assessment would be due to the formulation of the evaluated products.

### 3.2. ET Assessment

**Figure 2:**
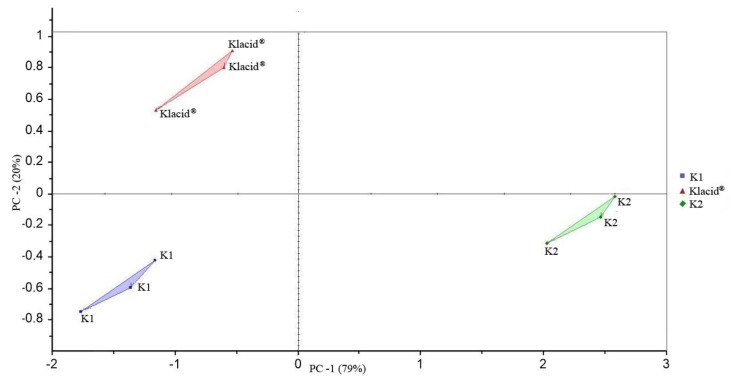
In vitro taste evaluation of clarithromycin suspensions using electrical tongue and principal component analysis (PCA).

Three products were included in this study. A principal component analysis (PCA) showed a very clear discrimination of the samples as shown in Figure 2. Two principal components were sufficient for describing the total variation of the data. The first principal component (PC1) explained 79.4% and the second principal component (PC2) explained 20.4%. The two PCs explained about 100%.

### 3.3. In Vivo Taste Assessment

#### Demographic Analysis

**Figure 3:**
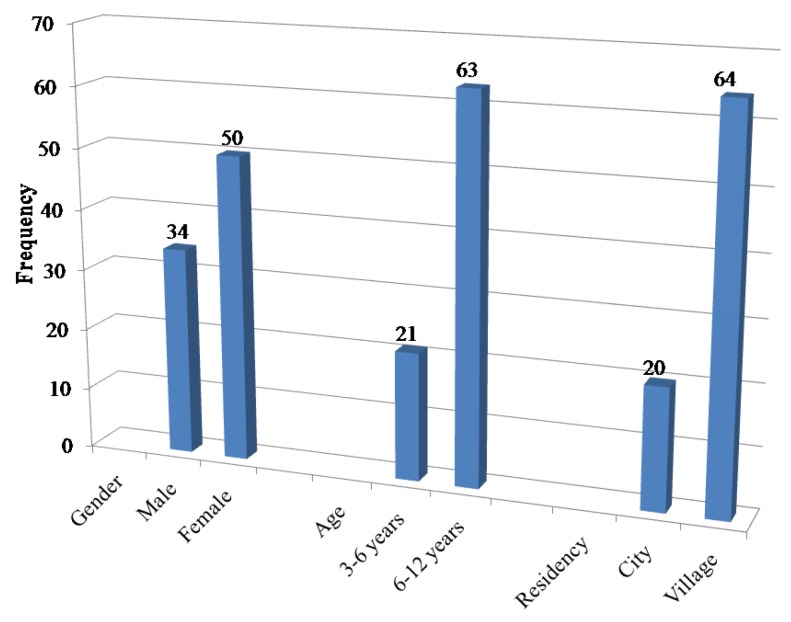
Demographic characteristics of the sample.

A total of 84 pediatric patients were involved in terms of baseline demographic and clinical characteristics as shown in Figure 3. The mean age was 8 ± 2 years. About half of the patients were females (59.5%), the age ranged from three years to 12 years. The majority of them had no other diseases. They were prescribed CM suspension (150 m/5 mLs) to treat infections that affect the upper and lower respiratory tract, skin, and soft tissue.

**Figure 4:**
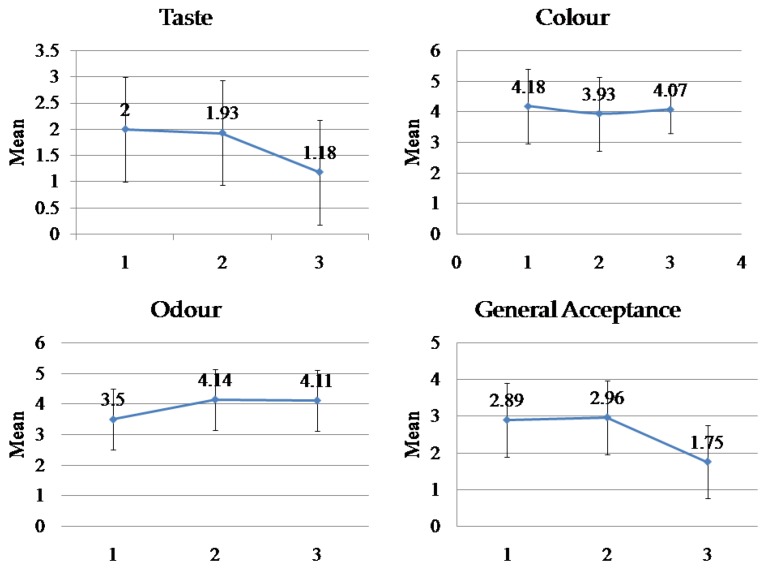
In vivo pediatric evaluation of Clarithromycin (CM) (125 mg/5 mL) suspensions. As the data were not normally distributed, the Kruskal–Wallis test was used to compare medians; the results of the three products are summarized in Table 2.

Concerning the results of the taste perception, Figure 4 shows the means ± SD of children’s perception according to the numerical average (one to five) from the hedonic scale.

Concerning the results of ranking taste and general acceptance by pharmacists, about 95% revealed that Klacid^®^ (125 mg/5 mLs) had better taste and was more accepted by paediatric patients than K1 (125 mg/5 mLs), while the generic K2 (125 mg/5 mLs)was the third in this order. This evaluation was in agreement with the number of prescriptions, as Klacid^®^ was the most commonly prescribed alternative 51.2% versus 41.5% for K1 and 7.3% for K2.

## 4. Discussion

The quality control results of the investigated CM suspensions demonstrated no significant difference in term of the assay or physical defects. In fact, the only significant difference was in the colour of these products, which does not affect the taste of the suspension. This would indicate that the observed differences in taste are mainly due to the differences in the formulations between Klacid^®^, Kl, and K2.The human tongue recognizes the basic tastes such as bitter, salt, umami, sweet, and sour. These tastes could be tested using the ET in order to differentiate between the tastes of food and pharmaceutical products. In fact, the ET is becoming an important tool in pharmaceutical development to assess the final taste of oral pharmaceutical products. Recently, a variety of studies were conducted on the ET and bioelectronic tongue to assess their ability in this field [35,36]. Moreover, the ET calculates the % pattern of discrimination between the tested samples. This index takes into account the difference between the centers of gravity and dispersion of each group. The closer the index to 100%, the greater the distance between the centres of gravity and the smaller the dispersion within groups (Alpha MOS, 2009). The discrimination index (Di) gives the discrimination quality through an indication of the surface between the groups as reported in Equation (1) and (2), respectively. In fact, when groups are distinct, the Di is calculated according to Equation (1), while when groups overlap, the discrimination index is calculated according to Equation (2).

Di = 100 * [1 − [(Surface (A) + Surface (B) + Surface(C))/(Total Surface)]](1)

Di= −(Σ Intersection Surface/Total surface) * 100(2)

The evaluation of the taste by ET revealed a significant difference between these products. Table 1 showed the results of a similarity test to evaluate the difference among different samples. The latter test describes three parameters, i.e., distance *p*-value and pattern discrimination index (%). The higher the distance between samples, the higher the difference in the taste between the assessed samples (Figure 2 and Table 1). Moreover, the ET calculates the % pattern of discrimination between the tested samples. This index takes into account the difference between the centers of gravity and dispersion of each group. The closer the index to 100%, the greater the distance between the centers of gravity and the smaller the dispersion within groups (Alpha MOS, 2009). As can be seen from the results in Figure 2 and Table 1, the distance between the groups was different. In fact, the distance between Klacid^®^ and K1 was 0.64, while between Klacid^®^ and K2 it was 3.12 and between K1 and K2, it was 2.78. This may indicate a higher taste similarity between the first two products (Klacid^®^ and K1) than between Klacid^®^ and K2^®^. Moreover, the pattern discrimination indexes between (K1 and Klacid^®^), (K1 and K2 and (Klacid^®^ and K2) were 8.81%, 65.75%, and 71.94%, respectively, which suggests that K1 and Klacid^®^ are the most similar medicines in term of taste, since they had the shorter distance and a lowest % index of discrimination pattern. All these comparisons were statistically significant since the *p*-values were <0.001 in all cases.

As the main goal of this work was to verify the ability of the ET to evaluate the taste of oral formulations, we further carried out in vivo evaluation of the three suspensions. Accordingly, the in vivo evaluation was conducted on paediatric patients as the ultimate target population. 

The obtained results concerning the taste and general patient acceptance, to some extent, confirmed those obtained by the ET. A significant difference was observed between these three products in terms of taste and the general acceptance by pediatricpatients (*p*-value < 0.05 (Figure 4 and Table 2). The highest score was for Klacid^®^, then K1followed by K2.

In addition, the order of taste (from the best to the least) was also confirmed by community pharmacists who get their experience from the general feedback of patients and their parents, as they ranked Klacid^®^ at the top of this classification. This may indicate a positive role of the ET in the taste assessment of drug products. Therefore, an ET can be used as a suitable, sensitive, economic and safe tool to assess the taste and general acceptance of oral drugs, especially in paediatric patients who are a vulnerable category of patients and who need to be safeguarded from any harmful effect of clinical trials.

## 5. Conclusions

Our results clearly showed a significant difference in term of taste between the evaluated brand (Klacid) and two generics in the Palestinian market. Klacid showed superiority in terms of taste, as evident from the ET and in vivo analysis. Moreover, our results confirmed the capability of an alpha-astree ET in the taste assessment of oral suspensions and in predicting volunteer responses, which highlights its beneficial use as an in vitro taste assessment tool and as an alternative to human-based taste evaluations.

## Figures and Tables

**Table 1:** Similarities between groups of K2, Klacid^®^ and K1 using the similarity function of the groups.

Number	Product Name	Reference	Distance	*p*-Value	Pattern Discrimination Index (%)
1	K1	Klacid^®^	0.64	<0.001	8.81
2	K1	K2	2.78	<0.001	65.75
3	Klacid^®^	K2	3.13	<0.001	71.94

**Table 2:** Comparison between the three products based on in vivo pediatric evaluation of CM (125 mg/5 mL) suspensions. (Kruskal Wallis test)

Variable	Klacid Median (Q1–Q3)	K1 Median (Q1–Q3)	K2 Median (Q1–Q3)	*p*-Value
Colour	4.5 (4–5)	4 (3–5)	4 (4–5)	0.382
Smell	4 (3–5)	4.5 (3–5)	4 (4–5)	0.209
Taste	1.5 (1–3)	1 (1–3)	1 (1–1)	0.011
General acceptance	3 (1–4)	3 (2–3.75)	1.5 (1–2.75)	0.001
